# The Experience of Stigma in People Affected by Fibromyalgia: A Metasynthesis

**DOI:** 10.1111/jan.16773

**Published:** 2025-01-21

**Authors:** Benedetta Colombo, Eleonora Zanella, Alessandro Galazzi, Paola Arcadi

**Affiliations:** ^1^ Department of Biomedical Sciences for Health University of Milan Milan Italy; ^2^ Department of Healthcare Professionals ASST Melegnano‐Martesana Milan Italy; ^3^ Department of Medicine University of Udine Udine Italy

**Keywords:** chronic pain diseases, fibromyalgia, invisible disease, qualitative research, quality of life, stigma

## Abstract

**Aim:**

To review the qualitative literature regarding how people with fibromyalgia experience and are impacted by stigma.

**Design:**

A systematic review and metasynthesis of qualitative studies was conducted following the Thomas and Harden method.

**Methods:**

The electronic databases PubMed, CINAHL, PsycInfo, Embase and Scopus were queried (September 2023). No publication year limit was set. Twelve studies were included in the final analysis. The findings were reported according to the Enhancing Transparency in Reporting the Synthesis of Qualitative Research (ENTREQ) statement.

**Findings:**

Three main themes with 11 sub‐themes were identified: manifestations and roots of stigma, avoidance and coping strategies, and consequences of stigma. Stigma arises from disbelief due to the invisibility of symptoms, protracted time to diagnosis and gender stereotyping, especially against women. Various strategies to avoid or cope with prejudice may involve social isolation, hiding the disease, controlling information, getting closer to or further away from other patients, acknowledging and understanding the disease. Stigmatisation can diminish a person's integrity and dignity, undermine trust in health care professionals and worsen suffering.

**Conclusion:**

The metasynthesis findings align with previous research highlighting the pervasive stigma associated with chronic pain conditions. Greater awareness of the impact of disease‐related stigma on individuals with fibromyalgia is crucial, not only among health care professionals but also within broader societal and institutional contexts.

**Implications for the Profession and/or Patient Care:**

Understanding the stigma experienced by individuals with fibromyalgia can guide health care professionals in adopting more empathtic approaches, potentially improving the diagnostic process and the overall management of the condition.

**Impact:**

This study highlights the profound impact of stigma on individuals with fibromyalgia, emphasising the need for greater awareness and targeted interventions to address stigma in clinical practice and societal contexts.

**Patient or Public Contribution:**

No patient or public contribution.

**Summary**

- What does this paper contribute to the wider global clinical community?
  ○ It highlights how stigma arises in individuals with fibromyalgia, focusing on its roots in disbelief, diagnostic challenges and gender stereotyping.
  ○ It provides insights into how patients manage or cope with stigma through strategies such as social isolation, hiding the illness and selective disclosure.
  ○ It emphasises the consequences of stigma, including diminished dignity, erosion of trust in health care professionals, and increased physical and psychological suffering.
  ○ It underscores the importance of fostering awareness among health care professionals to recognise and address stigma, thereby improving care pathways and patient outcomes.

## 1 Introduction

Stigma, a phenomenon intrinsically linked to social perception, manifests as a mark of shame or dishonour that leads to social exclusion and discrimination (World Health Organisation 2020). It is a highly discrediting attribute that transforms the individual from a complete and integrated person to a marginalised individual (Goffman 1986). The consequences of stigma are profound and varied: lower self‐esteem, difficulty in social relationships, social isolation, problems at work and lack of understanding from friends, family and colleagues (Borenstein 2020).

In health care, stigma often manifests in conditions perceived as being dangerous, incurable and contagious. Studies have underscored how stigma can diminish adequate care (Alonzo and Reynolds 1995; Earnshaw and Chaudoir 2009). Furthermore, the impact of stigma extends beyond care access to encompass various aspects of patient well‐being, including psychological distress, social isolation and reduced quality of life (Corrigan 2016; Sirey et al. 2001; Prosperi and Bille 2020). Chronic pain is also subject to stigma, as patients are often not believed and are mistaken for ‘imaginary patients’ (Goldberg 2010; Perugino et al. 2022). This stigma is particularly pronounced in fibromyalgia, a condition that affects 2%–4% of the world's population, predominantly women (Kocyigit and Akyol 2022).

Fibromyalgia is a chronic condition characterised by widespread musculoskeletal pain, often accompanied by fatigue, sleep disturbances, cognitive difficulties (commonly referred to as ‘fibro fog’), and emotional distress (Galvez‐Sánchez, Duschek, and Reyes Del Paso 2019). The condition is complex and multifactorial, with its exact cause remaining unclear. Fibromyalgia is often associated with heightened pain sensitivity due to abnormalities in pain processing pathways in the central nervous system (Galvez‐Sánchez, Duschek, and Reyes Del Paso 2019). These symptoms can severely impair quality of life, limiting patients' ability to perform daily activities and maintain social and professional roles (Kocyigit and Akyol 2022).

Diagnosing fibromyalgia presents significant challenges, as it involves navigating a complex array of symptoms without the guidance of definitive diagnostic biomarkers (De Ruddere and Craig 2016). This complex scenario often translates into frustration and disillusionment for patients when encountering disbelief by health care professionals, family and friends (Kocyigit and Akyol 2022). Furthermore, stigma can cast a shadow over every aspect of living with fibromyalgia. Misperception and misplaced judgement can lower one's self‐esteem and dignity, resulting in disability, depression and a sense of social isolation (Bean et al. 2022).

In such situations, patients often face the struggle to be believed while seeking relief of symptoms. The stigma associated with fibromyalgia reduces well‐being (Van Alboom et al. 2021). It is critical, therefore, that nurses understand the experience of stigma in patients with chronic pain, especially those with fibromyalgia (De Ruddere and Craig 2016). A better understanding of the illness and its management could help reduce stigma and improve overall well‐being (De Ruddere and Craig 2016). A comprehensive overview of the experience of stigma is currently lacking, however. Overall stigma profoundly impacts individuals with fibromyalgia by reducing their quality of life, undermining psychological well‐being, and straining the relationship between patients and health care professionals (Wasti et al. 2023). It can also hinder treatment adherence and create significant barriers to accessing appropriate care and the social support necessary for managing the condition. A deeper understanding of the stigma surrounding fibromyalgia is essential, not only to address the misconceptions and biases that perpetuate it but also to develop targeted intervention strategies aimed at both reducing stigma and improving the overall management and support for individuals with this condition.

## 2 Aim

This study aimed to review the qualitative literature regarding how people with fibromyalgia experience and are impacted by stigma.

## 3 Methods

### 3.1 Study Design

A systematic review with metasynthesis of qualitative studies was performed, employing the Thomas and Harden (2008) method, a widely used framework for thematic synthesis that involves three stages: coding the text, developing descriptive themes and generating analytical themes. The findings are reported according to the Enhancing Transparency in Reporting the Synthesis of Qualitative Research (ENTREQ) statement (Tong et al. 2012), which provides guidelines to improve the clarity and completeness of qualitative synthesis reporting.

The research protocol was registered with the University of Milan on 4 September 2023.

### 3.2 Research Strategy

The research question was: What is the impact of stigma on the illness experience of people with fibromyalgia? a comprehensive search for studies was conducted starting in September 2023 with a preliminary consultation of the Cochrane Library and Joanna Briggs Institute databases to identify systematic reviews. Subsequently, the PubMed, Cumulative Index to Nursing and Allied Health Literature (CINAHL), Embase, Scopus and Psycinfo databases were queried, as detailed in Table S1. The inclusion criteria comprised primary qualitative or mixed‐methodology studies in which qualitative analysis was conducted on the experience of adults with fibromyalgia, studies that fully or partially explored the experience of stigma in adults with fibromyalgia and studies published in English, Italian or Spanish. No publication year limit was set. Exclusion criteria were quantitative studies, mixed‐methods studies with only quantitative analysis and studies involving children.

### 3.3 Study Selection

**FIGURE 1:**
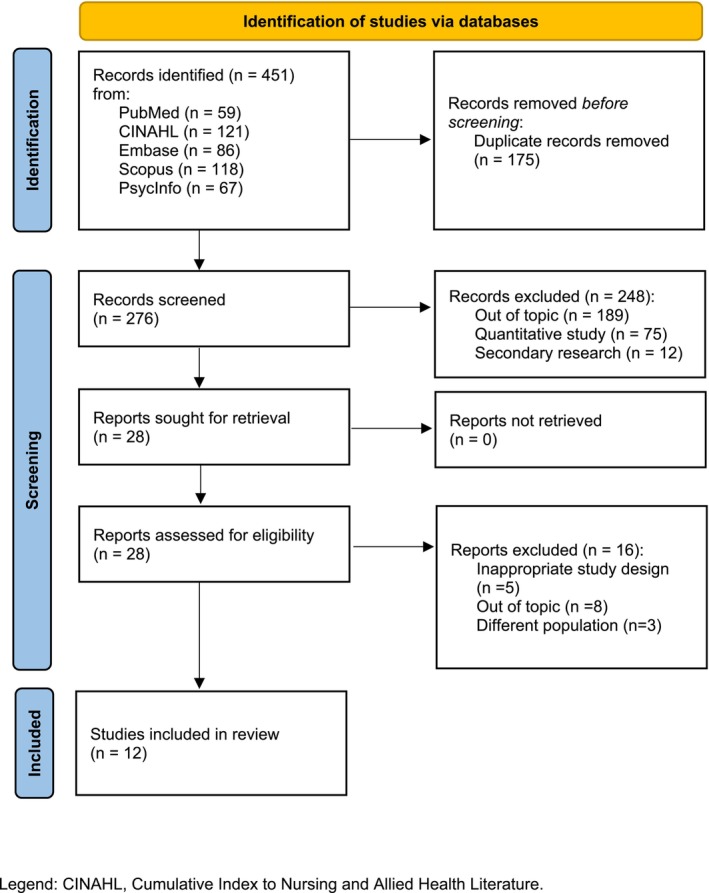
PRISMA flow diagram.

Studies were selected following PRISMA guidelines (Hutton et al. 2015). The search string retrieved 451 records, which were then uploaded on the Rayyan platform (Ouzzani et al. 2016). After the removal of duplicates, 276 records remained. Blind screening of titles, abstracts and full texts was carried out by two authors (BC and EZ). A third author (PA) was consulted to resolve disagreement. Three studies were excluded because they involved a population sample different from that of our study, five because of inappropriate study design, and eight because the topic was irrelevant to our study. Twelve studies met the criteria and were included (Figure 1) (Page et al. 2021).

### 3.4 Data Extraction

The study results were reported in a data extraction table that included the following fields: author, year of publication, country, purpose, study type, context, sample, data collection methods, data analysis methods, and identified themes and subthemes. The data extraction table was piloted with three studies and no modifications were deemed necessary. Two authors (BC and EZ) performed the data extraction, while a third author (PA) verified the results and resolved disagreement.

### 3.5 Quality Assessment

Each study was assessed following the Joanna Briggs Institute critical appraisal checklist for qualitative research to determine its quality and methodology (Lockwood, Munn, and Porritt 2015). The checklist consists of 10 questions, each of which can be answered ‘yes’ or ‘no’ or ‘unclear’. Two authors (BC and EZ) independently assessed study quality, while a third author (PA) resolved disagreement. Table S2 describes the quality assessment scores for all studies. The metasynthesis included six studies scored 8/10 (Cunningham and Jillings 2006; Escudero‐Carretero et al. 2010; Rodham, Rance, and Blake 2010; Juuso et al. 2011; Henriksson 1995; Paulson, Norberg, and Danielson 2002), three studies scored 9/10 (Armentor 2017; Asbring and Närvänen 2002; Taylor et al. 2016), and three studies scored 10/10 (Peres 2021; Russell et al. 2018; Söderberg, Lundman, and Norberg 1999). Most issues concerned reflexivity bias, specifically the influence of the researcher on the study and vice versa.

### 3.6 Data Synthesis

For this study, we applied the Thomas and Harden method of thematic and content analysis for the synthesis of qualitative studies (Thomas and Harden 2008). The themes were derived inductively. Two researchers (PA and EZ) independently read each study and extracted participant quotes to gain a preliminary understanding of the topic. Initially, the study findings were coded inductively based on the meaning of the text. For studies with broader aims around the lived experience of fibromyalgia, rather than stigma specifically, we focused on extracting data that explicitly or implicitly addressed stigma‐related aspects. This was achieved by identifying codes and concepts associated with social judgement, disbelief and discrimination reported by participants. By refining the analysis to align with the study's focus on stigma, broader themes were distilled into specific concepts related to the stigmatising experience. To create themes and sub‐themes, codes were systematically analysed and grouped based on patterns of similarity and distinctiveness. This process ensured that related concepts were categorised together while preserving meaningful differences. Themes and sub‐themes were then re‐checked against the findings from the primary studies to confirm their alignment and ensure they accurately represented the original data. Consensus validation revealed no discrepancies or disagreements between the two researchers. Finally, the themes were given a descriptive narration and illustrated with participant quotations. No software was used.

## 4 Results

### 4.1 Study Characteristics

Table 1 presents the characteristics of the 12 studies. The studies were conducted between 1995 and 2021. Five were conducted in Sweden (Asbring and Närvänen 2002; Henriksson 1995; Juuso et al. 2011; Paulson, Norberg, and Danielson 2002; Söderberg, Lundman, and Norberg 1999), two in the United States of America (Armentor 2017; Taylor et al. 2016), one in Canada (Cunningham and Jillings 2006), one in Spain (Escudero‐Carretero et al. 2010), one in Brazil (Peres 2021), one in England (Rodham, Rance, and Blake 2010), and one in Germany (Russell et al. 2018). The main objectives were to: describe the experience of fibromyalgia symptoms in everyday life (Henriksson 1995; Juuso et al. 2011; Paulson, Norberg, and Danielson 2002; Russell et al. 2018), narrate the experience of illness (Cunningham and Jillings 2006; Rodham, Rance, and Blake 2010; Söderberg, Lundman, and Norberg 1999; Taylor et al. 2016), investigate the perception of illness in collective imagination (Peres 2021), learn about expectations from the health care system (Escudero‐Carretero et al. 2010), examine the perception of stigma and use of avoidance strategies (Asbring and Närvänen 2002), and explore relational approaches to manage interpersonal relationships, including those with health care professionals (Armentor 2017).

**TABLE 1:** Study Characteristics.

Author Year Country	Aim	Design	Setting	Sample method	Data collection	Data analysis	Themes and sub‐themes
Armentor (2017) USA	To explore the ways women diagnosed with fibromyalgia manage relationships with family, friends, colleagues, and health care professionals	Descriptive qualitative	Not stated	Convenience then snowball sampling; sample size 20 women aged 32–80 years	In‐depth semi‐structured interviews (60 min on average)	Grounded theory modified approach with generation of descriptive categories and core concepts	Three themes and five sub‐themes: a contested and stigmatised illness (lack of understanding; disbelief); approaches to communication (awareness of pain; comparison with other illnesses; being direct); avoidance as an approach for managing stigma
Asbring and Närvänen (2002) Sweden	To determine whether women experience fibromyalgia‐related stigma and how they avoid stigmatisation	Grounded theory	Outpatient clinics and homes	Purposeful sampling; sample size 25 women, 12 with chronic fatigue syndrome, 13 with fibromyalgia, aged 32–65 years	Semi‐structured interviews (60–150 min)	Preliminary analysis in Grounded Theory approach; analysis in seven stages, with generation of themes and sub‐themes	Two themes and eight sub‐themes: stigmatising aspects (questioning morality; psychologizing symptoms; diagnoses as relief and burden; strategies to manage stigma); strategies to handle stigma (keeping a distance from others; hiding; spreading or withholding information; withdrawing or approaching other patients)
Cunningham and Jillings (2006) Canada	To explore and describe the lives of persons with fibromyalgia	Interpretive descriptive	Not stated	Purposeful sampling; sample size one man and seven women; age not stated	In‐depth, semi‐structured interviews (45–120 min)	Comparative inductive analytic method	Two themes: living with the symptoms of fibromyalgia; management of fibromyalgia
Escudero‐Carretero et al. (2010) Spain	To explore what persons with fibromyalgia experience and what they expect from the health care system and health care providers	Descriptive qualitative	Patient associations	Convenience telephone sampling with stratification; sample size 21 persons with fibromyalgia, 20 women and 1 man aged 33–62 years	Focus groups with semi‐structured interviews (120–180 min)	Qualitative content analysis in four stages: (1) careful reading of the transcriptions; (2) definition of the analysis categories (code tree) based on the objectives of the study and the information obtained; (3) assigning codes to text fragments and (4) interpreting, systematising and discussing the results	Two themes and six sub‐themes: experience of fibromyalgia (worsening quality of life; misunderstanding and disbelief; pain and uncertainties); expectations toward the healthcare system and its operators (help in living with this disease; continuity of care; need for time and comprehension)
Henriksson (1995) Sweden and USA	To compare how women in two different countries perceive their life with fibromyalgia	Descriptive qualitative	Hospital outpatient clinics	Purposeful sampling with participant selection in a university rheumatology hospital; sample size 40 Caucasian women, 20 residing in the USA and 20 in Sweden	Semi‐structured interviews based on questions from the Occupational Case Analysis Interview and Rating Scale assessment (35–55 min)	Qualitative content analysis; data coding and analysis using the Ethnograph computer program	Three themes and 14 sub‐themes: reception by the health care system (anxiety; disbelief; relief; reactions of others); conflict between appearance and ability (mistrust and doubt; family relationships; understanding and support; not identifying); consequences (limitation of motor performance; physical effort increases pain; loss of ability; lack of physical fitness and loss of youth; consumption of time; loss of future)
Juuso et al. (2011) Sweden	To deetermine the significance of pain for women diagnosed with fibromyalgia	Hermeneutic phenomenological qualitative	Home, patient associations	Convenience sampling; sample size 15 women aged 38–64 years	Narrative interviews (45–115 min)	Phenomenological hermeneutic interpretation of interview texts; data analysed in three stages: naive understanding; structural analysis; and global understanding	Two themes and five sub‐themes: experiencing a reluctant body (living with an invisible change in the body; feeling forced into an unfamiliar body; trying to endure an overwhelming feeling of unpredictability); experiencing a good life despite everything (finding relief through distraction; feelings of reconciliation)
Paulson, Norberg, and Danielson (2002) Sweden	To describe how men living with fibromyalgia‐type pain experience being patients	Descriptive qualitative	Interviews conducted in patient's home, researcher's office or by telephone according to patient preference	Sample selected from patients admitted to a rheumatology hospital in central Sweden; sample size 14 patients aged 41–56 years	Narrative interviews (40–120 min)	Qualitative content analysis; text divided into health care areas; areas then analysed to create coherent categories and identify themes	Five themes: fear of being considered a crybaby; feeling like a guinea pig; feeling confident; feeling neglected; not feeling healed
Peres (2021) Brasil	To investigate fibromyalgia in women	Qualitative study based on the psychoanalytic investigative method	Group interviews conducted in a private room of a non‐governmental organisation	Convenience sample	Group interviews (about 90 min) based on the Drawing‐Story with Theme Procedure	Data analysis guided by technical movements recommended by Herrmann for operationalization of the psychoanalytic investigation method	Two themes and three sub‐themes: a body that hurts (change of life; impotence of illness); added pain (invisibility, misunderstanding, stigmatisation)
Rodham, Rance, and Blake (2010) UK	To compare the experiences of those with fibromyalgia and their spouses	Qualitative phenomenological	Patient's home	Local fibromyalgia support group members and their spouses interviewed about their experience; sample size four women diagnosed with fibromyalgia aged 43–65 years and their husbands aged 38–59 years	Semi‐structured interviews (30–60 min)	Interpretative phenomenological analysis according to Smith and Osborn	One theme and three sub‐themes: loss of identity (attitudes of others; invisible illness; change of role)
Russell et al. (2018) Germany	To explore the perception of fatigue, sleep dysfunction, and exercise in persons with fibromyalgia	Descriptive qualitative	Not stated	Purposive sampling from patient support groups that engaged in therapeutic exercise for managing their condition; sample size 12 women and 2 men; age not stated	Three focus groups with semi‐structured interviews (60–90 min)	Data subjected to thematic content analysis; the themes derived from coding, categorising, discussion, and reflection	One theme and two sub‐themes: lack of understanding (sense of loss; impact of physical and psychological symptoms)
Söderberg, Lundman, and Norberg (1999) Sweden	To understand the meaning of women's experience of living with fibromyalgia	Hermeneutic phenomenological qualitative	Interviews before starting treatment in an outpatient clinic	Convenience sampling, women with fibromyalgia invited by letter; sample size 14 women aged 35–50 years	Guided narrative interviews (15–60 min)	Ricoeur's phenomenological‐hermeneutic method applied to analyse and interpret the interview texts	Three themes and eight sub‐themes: loss of freedom (a body that suffers and other bodily sensations; fatigue, and loss of energy; living a changed daily life; economic restrictions); threat to integrity (loss of credibility and invisibility of the disease; lack of knowledge about fibromyalgia and society's negative attitude); struggling to gain understanding and relief (seeking explanations and understanding; seeking relief and planning one's daily life in accordance with the disease)
Taylor et al. (2016) USA	To document the experiences and the meaning‐making reported by persons with fibromyalgia	Descriptive qualitative	Home in a rural setting in a Mid‐Atlantic state of USA	Convenience sampling; participants learned about the study from a website of ongoing clinical trials conducted at the medical center and from study brochures distributed to persons interested in participating; sample size 19 women and 1 man aged 49.1 years on average	Semi‐structured interviews (40–60 min)	Qualitative content analysis to identify recurrent or common themes and to group the data into meaningful units	Five themes: loss; fear and uncertainty; impact of stress; stigma associated with fibromyalgia; coping with courage

Abbreviations: UK, United Kingdom; USA, United States of America.

Most of the studies used a qualitative methodology with a descriptive (Cunningham and Jillings 2006; Escudero‐Carretero et al. 2010; Henriksson 1995; Paulson, Norberg, and Danielson 2002; Peres 2021; Russell et al. 2018; Taylor et al. 2016) or a phenomenological approach (Juuso et al. 2011; Rodham, Rance, and Blake 2010; Söderberg, Lundman, and Norberg 1999). Two studies used the Grounded Theory approach (Armentor 2017; Asbring and Närvänen 2002) and one study used a psychoanalytic investigative method (Peres 2021).

Six of the 12 studies collected data via semi‐structured interviews (Armentor 2017; Asbring and Närvänen 2002; Cunningham and Jillings 2006; Henriksson 1995; Rodham, Rance, and Blake 2010; Taylor et al. 2016), with focus groups in two studies (Escudero‐Carretero et al. 2010; Russell et al. 2018). Two studies employed in‐depth narrative interviewing (Juuso et al. 2011; Paulson, Norberg, and Danielson 2002). Finally, one study was based on the thematic story‐drawing method with subsequent interviews (Peres 2021).

In total, the 12 studies involved 206 patients with fibromyalgia (182 women and 24 men, age range 32–80 years). Study participants were recruited from outpatient clinics (Asbring and Närvänen 2002; Henriksson 1995; Söderberg, Lundman, and Norberg 1999), patient associations or organisations (Escudero‐Carretero et al. 2010; Juuso et al. 2011; Peres 2021), hospitals (Paulson, Norberg, and Danielson 2002), public places of rural living environments (Armentor 2017; Russell et al. 2018; Taylor et al. 2016) or at home (Juuso et al. 2011; Rodham, Rance, and Blake 2010).

### 4.2 Metasynthesis

Three main themes and 11 subthemes regarding the experience of stigma were identified (Table 2). The three main themes were: (1) manifestations and roots of stigma, (2) avoidance and coping strategies, and (3) consequences of stigma.

**TABLE 2:** Themes and sub‐themes from qualitative analysis (metasynthesis) of the studies.

Themes	Sub‐themes	Armentor 2017	Asbring and Närvänen 2002	Cunningham and Jillings 2006	Escudero‐Carretero et al. 2010	Henriksson 1995	Juuso et al. 2011	Paulson, Norberg, and Danielson 2002	Peres 2021	Rodham, Rance, and Blake 2010	Russell et al. 2018	Söderberg, Lundman, and Norberg 1999	Taylor et al. 2016
Manifestations and roots of stigma	Disbelief due to the invisibility of the symptoms of the disease	x	x	x	x	x	x	x	x	x	x	x	x
	Doubt and error in diagnosis	x	x	x	x	x		x		x	x	x	x
	Debasement of the disease as a gender stereotype	x	x										
Avoidance and coping strategies	Social isolation	x	x				x	x					x
	Hiding the disease	x	x			x			x	x			x
	To tell or not to tell	x	x			x	x	x	x				x
	Getting closer to or further away from other patients		x	x	x							x	x
	Acknowledging and understanding the disease	x	x	x	x	x		x			x	x	x
Consequences of stigma	Threat to integrity and dignity	x	x	x	x	x	x	x	x	x	x	x	x
	Loss of trust in healthcare professionals	x	x	x						x			
	Increased suffering	x	x	x	x	x	x		x		x	x	x

#### 4.2.1 Manifestation and roots of stigma

Manifestation and roots of stigma referred to what individuals with fibromyalgia consider stigmatising, as well as the perceived reasons or societal beliefs underlying the stigmatisation of their illness. This theme was further divided into three subthemes: disbelief due to the invisibility of the symptoms of the disease, doubt and error in diagnosis and debasement of the disease as a gender stereotype.

##### 4.2.1.1 Disbelief due to the Invisibility of the Symptoms of the Disease

The persons with fibromyalgia reported that others do not believe the veracity of their experience. One of the causes of disbelief is the lack of visible signs of illness, for example, ‘Others can't understand what they cannot see.’ (Juuso et al. 2011).

The experience of pain, chronic fatigue, muscle tension and other symptoms attributable to fibromyalgia is questioned by health care professionals, colleagues, family members and friends (Armentor 2017; Asbring and Närvänen 2002; Cunningham and Jillings 2006). Furthermore, because they generally seem well, their condition is often doubted (Escudero‐Carretero et al. 2010; Paulson, Norberg, and Danielson 2002). For example, ‘You don't look sick, you can't be sick.’ (Söderberg, Lundman, and Norberg 1999).

The variability, unpredictability, and difficulty in describing signs and symptoms of illness all weaken patients' credibility (Henriksson 1995; Juuso et al. 2011). Indeed, they often described the difficulty family, friends, and colleagues have in understanding disease‐related limitations, including the need to rest more and the difficulty with lifting objects and carrying out activities considered normal by society (Armentor 2017; Asbring and Närvänen 2002; Cunningham and Jillings 2006; Escudero‐Carretero et al. 2010; Paulson, Norberg, and Danielson 2002; Peres 2021; Rodham, Rance, and Blake 2010; Russell et al. 2018; Söderberg, Lundman, and Norberg 1999; Taylor et al. 2016). For instance, ‘It's very difficult because you look fine. You don't—nobody sees that there's anything wrong with you. It's hard for them to accept that you do have this difficulty. If you have a broken leg, and it's in a cast, then your family doesn't expect you to get up, and clean house, and do dishes, and run five miles. When you look perfectly healthy, it's hard for them to accept that there's anything wrong with you.’ (Armentor 2017).

##### 4.2.1.2 Doubt and Error in Diagnosis

The often protracted time between symptom onset and obtaining a diagnosis was cited as a contributing factor to stigma (Armentor 2017; Asbring and Närvänen 2002; Cunningham and Jillings 2006; Escudero‐Carretero et al. 2010; Henriksson 1995; Paulson, Norberg, and Danielson 2002; Rodham, Rance, and Blake 2010; Russell et al. 2018; Söderberg, Lundman, and Norberg 1999; Taylor et al. 2016). Health care professionals often interpreted the symptoms reported by patients as imaginary or relatively unimportant. This disbelief leads to a frequent misdiagnosis of psychiatric or psychological disorders, which patients perceive as highly stigmatising (Paulson, Norberg, and Danielson 2002; Rodham, Rance, and Blake 2010; Russell et al. 2018; Söderberg, Lundman, and Norberg 1999), as expressed by two women, ‘Because everybody kept saying, “Oh nothing's wrong. You're depressed.” I kept getting, “You're depressed. You're depressed.” I was like, “No.”’ (Armentor 2017), ‘I felt… like the doctor said I was a hypochondriac… or crazy…’ (Henriksson 1995). Patients often cite the health care professionals' lack of knowledge of fibromyalgia as the main cause of stigma. During the course of treatment, health care professionals often tend to question the validity of the diagnosis, raising further doubts and uncertainties (Cunningham and Jillings 2006; Escudero‐Carretero et al. 2010). For example, ‘I went to many different doctors and they simply told me “You have a back injury, go back to work, it will get better.” Every time I went back to work I got much worse, and even after I found out what it was, they still didn't believe I was sick.’ (Henriksson 1995).

##### 4.2.1.3 Debasement of the Disease as a Gender Stereotype

Perceived bias stems from people's associating the illness predominantly with women, given its higher prevalence among females. This bias leads to questions and judgements rooted in gendered stereotypes, such as the belief that women are more likely to exaggerate their symptoms or that their complaints are emotionally driven rather than medically valid (Armentor 2017). The perception of female fragility, linked to an assumed greater concern about the emotional sphere and the self‐care inherent to the traditional female role, generates uncertainties about the veracity of symptoms and the clinical causes before a diagnosis is made. For example, ‘They thought I was crazy’ (Asbring and Närvänen 2002), ‘they told me, “Oh, it's all in your head.”’ (Armentor 2017).

These perceptions persist even after a diagnosis is confirmed. Women are often perceived as exaggerating rather than downplaying their concerns about symptoms. (Armentor 2017; Asbring and Närvänen 2002). For example, ‘Now that you know, you should feel better.’ (Asbring and Närvänen 2002).

#### 4.2.2 Avoidance and coping strategies

The second main theme was avoidance and coping strategies, that is, the ways that people diagnosed with fibromyalgia try to avoid, manage and ward off stigma. Five subthemes were identified: social isolation, hiding the disease, to tell or not to tell, getting closer to or further away from other patients, acknowledging and understanding the disease.

##### 4.2.2.1 Social Isolation

Social isolation is a common stigma avoidance strategy. Some patients related that they limit socialisation to avoid feeling judged by others. In addition, fatigue and chronic pain accompany this condition. In general, the patients reported avoiding interacting with other people. The reasons for this behaviour were fear of being judged, general disinterest in the condition, and lack of support and understanding (Armentor 2017; Asbring and Närvänen 2002; Juuso et al. 2011; Paulson, Norberg, and Danielson 2002; Taylor et al. 2016). For example, ‘My girlfriend the other day called and said “You wanna go to the softball tournament on Saturday?” I was like “Yeah, that'd be fun.” She was like “I figured you'd like to see some people since you haven't seen them in so long.” I'm like “Yeah, that'd be good.” Then panic. Well no, they can't see me like this. I can't—I don't want to talk to them. I don't want to have to explain everything to 25 different people.’ (Armentor 2017).

##### 4.2.2.2 Hiding the Disease

Another way to lighten the burden of stigma is to hide one's illness. By keeping a facade, patients can conform to others' expectations. Some patients reported that they maintain social relationships by playing the role of a happy, healthy, and normal individual, until they break down when alone at home. This strategy allows them to minimise prejudice against their real condition (Armentor 2017; Asbring and Närvänen 2002; Henriksson 1995; Peres 2021; Rodham, Rance, and Blake 2010; Taylor et al. 2016). For instance, ‘You don't want people to see that you are sick, for me it is important to feel beautiful on the outside, you know? Because my inside is not beautiful.’ (Henriksson 1995), ‘I can admit that there are a lot of my acquaintances who don't know that I… I've not told them about it. They believe that I am still working full‐time and that everything is as usual.’ (Asbring and Närvänen 2002).

##### 4.2.2.3 To Tell or Not to Tell

Very different avoidance strategies are enacted. Some patients chose to control information by talking only with trusted individuals about symptoms and their experience of the disease. Others, at the opposite end of the behavioural spectrum, openly faced the disease. They circumvented stigma by clearly stating their disease‐related needs and limitations. For example, ‘No, I prefer not to say so much, I was mocked at work when I had a hard day and had a lot of pain under my feet. It's like walking on glass actually and I had taken with me a paper about the pain so I could explain and then she came [the boss] and saw the paper and mocked me about it and that's why I've backed off and I don't want to tell anyone about the pain.’ (Juuso et al. 2011). ‘To my children who are now adults, obviously, I speak to them just like I do my doctor, actually, in a lot of ways. I'm very direct about it and if I'm having a bad day it's I'm having a day and you need to understand what that means for me.’ (Armentor 2017).

Other strategies include comparing fibromyalgia with more well‐known illnesses to make the situation more understandable to others. Patients also use metaphors and imagery to describe the impact more tangibly and engagingly. These approaches allow them to avoid talking about more abstract symptoms (Armentor 2017; Asbring and Närvänen 2002; Henriksson 1995; Juuso et al. 2011; Paulson, Norberg, and Danielson 2002; Peres 2021; Taylor et al. 2016). For instance, ‘A lot of times whenever I try to explain it early on, I would explain it that I feel like I have the worst flu in the world, but I don't have the flu. That's just how I feel. Every part of the body just hurts, no matter what it is.’ (Armentor 2017).

##### 4.2.2.4 Getting Closer to or Further Away From Other Patients

Some patients reported that joining a fibromyalgia self‐help group was a negative experience (Asbring and Närvänen 2002; Cunningham and Jillings 2006). Constant confrontation with the suffering of others is likely to intensify the stigma already associated with the illness, making it difficult to detach oneself from negative perceptions. For example, ‘Being in a group was rather depressing because you found that there would be whiners who couldn't put up with any pain and didn't put themselves forth to do anything, and just complained and complained… and so… it wasn't a good idea for me anyway to be in a group. It pulled me down.’ (Cunningham and Jillings 2006).

For other patients, however, the experience was extremely valuable. Group participation offered an environment of empathetic understanding and listening, providing essential support and facilitating more positive living with the disease (Asbring and Närvänen 2002; Cunningham and Jillings 2006; Escudero‐Carretero et al. 2010; Söderberg, Lundman, and Norberg 1999; Taylor et al. 2016). For example, ‘I went to the rheumatology department and met a support group, I think that helped me a lot… because I heard other people with the same problems as me, so you don't feel alone.’ (Söderberg, Lundman, and Norberg 1999).

##### 4.2.2.5 Acknowledging and Understanding the Disease

In general, the patients stated it was important for them to know about the illness and from this knowledge develop a strategy for dealing with stigma. This awareness helped them to stand up for themselves and feel empowered about their experience (Söderberg, Lundman, and Norberg 1999).

Dissemination of information about the disease is crucial. Some patients stated that they made copies of articles about fibromyalgia and shared them with colleagues, friends and family members to help them understand the implications of the condition. For example, ‘It feels especially difficult when it concerns this type of pain because it has poor credibility and my boss found it most helpful receiving this information—it makes it easier to understand.’ (Paulson, Norberg, and Danielson 2002).

Others, however, reported encouraging those around them to attend educational meetings about the illness (Armentor 2017; Asbring and Närvänen 2002; Cunningham and Jillings 2006; Escudero‐Carretero et al. 2010; Henriksson 1995; Paulson, Norberg, and Danielson 2002; Russell et al. 2018; Söderberg, Lundman, and Norberg 1999; Taylor et al. 2016). ‘The more my daughter learns about it, the more supportive she becomes, so I always stimulate her.’ (Henriksson 1995).

#### 4.2.3 Consequences of stigma

The third theme was the physical and psychological repercussions of stigma. Three subthemes were identified: threat to integrity and dignity, loss of trust in health care professionals and increased suffering.

##### 4.2.3.1 Threat to Integrity and Dignity

The fact of not being considered truly ill is described as a profound violation of one's dignity. A discrepancy emerges between how patients define themselves and how they are defined by others. Patients experience a negative attitude and lack of understanding that makes them feel disrespected as human beings and diminished in moral integrity (Armentor 2017; Asbring and Närvänen 2002; Cunningham and Jillings 2006; Escudero‐Carretero et al. 2010; Taylor et al. 2016). For example, ‘They make me feel like a piece of trash.’ (Taylor et al. 2016).

Many patients reported feeling neglected because of an unprofessional approach by physicians (Taylor et al. 2016). Others, however, felt like guinea pigs for doctors experimenting with treatments and therapies, without receiving an adequate explanation of what the treatment involved or experiencing any real benefit from it. This was perceived as debasement of identity and loss of dignity (Armentor 2017; Asbring and Närvänen 2002; Cunningham and Jillings 2006; Escudero‐Carretero et al. 2010; Henriksson 1995; Juuso et al. 2011; Paulson, Norberg, and Danielson 2002; Peres 2021; Rodham, Rance, and Blake 2010; Russell et al. 2018; Söderberg, Lundman, and Norberg 1999; Taylor et al. 2016). For example, ‘You feel like a mess as a person, sinking, because you say to yourself, “Well if I have nothing, why am I so bad? How do I prove it? I don't have a document, I don't have an analysis to back it up. It's not life… I can't live.”’ (Escudero‐Carretero et al. 2010).

##### 4.2.3.2 Loss of Trust in Health Care Professionals

This subtheme addresses the loss of trust in health care professionals. Some of the reasons for distrust are undoubtedly the disdain patients experience when trying to receive a diagnosis, the disbelief toward symptoms, and being labelled with a psychiatric tag. They reported how difficult it was to find an active listener to their health problems. The lack of empathy and support from heath care professionals leads to a loss of trust. The long waiting lists and the lack of understanding symptoms and support result in postponing visits and relying on alternative medicine (Armentor 2017; Asbring and Närvänen 2002; Cunningham and Jillings 2006; Rodham, Rance, and Blake 2010). For example, ‘Went to the hospital, and I have never been so angered in my life when one of the nurses walked by and somebody asked, “What's wrong with this lady?” “Oh!… the usual… menopause.” We are called complainers. I have one document from one of the doctors that said that I have psychological problems. I simply told him that “If I need a psychologist, I will see a psychologist. I am here to ask you a medical question.” The medical profession does not take it seriously.’ (Cunningham and Jillings 2006).

##### 4.2.3.3 Increased Suffering

A direct cause of stigma is the increased physical and psychological suffering. Due to general ignorance, lack of understanding and support, and invisibility of symptoms, patients fall into depression and suffering worsens (Armentor 2017; Asbring and Närvänen 2002; Cunningham and Jillings 2006; Escudero‐Carretero et al. 2010; Henriksson 1995; Juuso et al. 2011; Peres 2021; Russell et al. 2018; Söderberg, Lundman, and Norberg 1999; Taylor et al. 2016).

For some patients, psychological suffering is defined as the pain that hurts more than physical pain and intensifies it. For example, ‘People don't believe you have fibromyalgia, it's an extra pain because you don't have anyone's support, not even a family member.’ (Peres 2021). ‘When we talk about the pain, people turn their noses up at it, that hurts us a lot, it hurts more than the pain we feel.’ (Peres 2021).

## 5 Discussion

This metasynthesis aimed to explore how people with fibromyalgia experience stigma, providing insights into its manifestations, coping strategies and consequences, to deepen understanding of the illness and its impact on individuals. Three themes emerged from the analysis: manifestation and roots of stigma, avoidance and coping strategies and consequences of stigma.

Regarding the manifestation and roots of stigma, patients reported that, due to the invisibility of their symptoms, they encountered disbelief from friends, family, acquaintances and health care professionals. This observation is shared by previous studies that found that chronic pain diseases are often considered to be invisible (Paul‐Savoie et al. 2018; De Ruddere and Craig 2016). Those unaffected react with uncertainty when pain does not manifest clinically (Lundin et al. 2023).

The diagnostic pathway is difficult to follow because health care professionals tend to downplay patients' reported symptoms, especially when diagnostic tests show nothing remarkable. Patients with chronic pain are known to feel they are not being listened to (Thomas 2000) and that clinical stigma derives from underestimation of suffering by health care professionals (Williams 2016) and from psychological tagging of pain, ultimately ending in ineffective treatment (Tait, Chibnall, and Kalauokalani 2009).

Stigma is also rooted in gendered stereotyping. Indeed, women reported encounters in which their symptoms were minimised or attributed to a presumed psychosomatic cause associated with female sex (Cunningham and Jillings 2006). This reflects a history of gender discrimination in women's care, such as the medical categorization of hysteria in the 19^th^ century (Barker 2005). While hysteria is no longer considered an illness, the phenomenon is still felt whenever women seek health care (Armentor 2017). And it is accompanied by the belief that, being more emotional than men, women seek help more frequently; therefore, their pain is given a psychological explanation to cloak disbelief (Newton et al. 2013).

The second theme concerns avoidance and coping strategies, which encompass the behaviours patients engage in to avoid, manage and reduce stigma. Strategies to manage stigma include deciding whether to disclose or keep one's health condition confidential from others or to hide it and be perceived as healthy by society (Joachim and Acorn 2000). In addition, having no diagnosis, patients are reluctant to discuss their pain with others (Clarke and Iphofen 2005). Studies report that in their effort to avoid scepticism and not be seen as a burden, those living with chronic pain often employ silence and isolation as their only weapon of defence, while bearing chronic illness with fear and insecurity (Kengen Traska et al. 2012). Persons with fibromyalgia tend to hide it as a way to control the disbelief and stigma associated with the illness (Sim and Madden 2008). Participation in self‐help groups was found to be beneficial for some patients (Kogstad 2009). However, while it can be an effective intervention in the early stages of chronic illness, the complaining by others may wear down a person's motivation to remain in the group (Raymond and Brown 2000).

Information and knowledge are key to reducing stigma and boosting the legitimacy of experience. Confirmation of a diagnosis of chronic pain is closely linked to personal credibility, as it legitimises the patient's experience of pain (Van Rysewyk et al. 2023). Similarly, a definitive diagnosis of fibromyalgia serves to validate the reality of the disease, helping to counter scepticism and reduce negative attitudes from others (Mengshoel et al. 2018).

The third and final theme was the consequences of stigma. Not being considered ill is experienced as a profound violation of dignity and an even heavier burden than the illness itself. A direct cause of stigma is the challenge to a person's sense of belonging, dignity and credibility due to disbelief of signs and symptoms (Werner and Malterud 2003). Patients who do not receive adequate medical attention, are not listened to or are continuously doubted experience a sense of exclusion that violates their dignity (Kleinman 1988).

Perceived stigma creates mistrust toward health care professionals. The main causes are the disdain encountered in seeking a diagnosis, disbelief of symptoms, and the labeling of the problem with a psychiatric tag. The literature on this issue in the context of chronic pain illness is scant. When patients sense disbelief from health care professionals, they may feel frustrated or angry. If health care professionals accept and show empathy toward the pain experience, they might be able to gain a patient's trust and reduce the attached stigma and diffidence (Newton et al. 2013).

Due to the lack of understanding and support and the invisibility of symptoms, patients experience physical and psychological suffering. Indeed, disbelief toward chronic pain increases emotional distress (Newton et al. 2013). In addition, the lack of obvious physical signs that may explain the pain and the feeling of not being taken seriously, especially by health care professionals (Maiandi et al. 2021), lead to distress and depression (Clarke and Iphofen 2008).

The findings of this metasynthesis indicate that stigma has an extremely negative impact on all aspects of a person's life. Disease‐related stigma is a powerfully dehumanising phenomenon (Pettit 2008) that reduces people to stereotypes and undermines their dignity and self‐esteem.

## 6 Implications for Practice

This study offers insights for clinical practice by addressing the stigma experienced by people with fibromyalgia. It highlights the importance of recognising the roots and consequences of stigma and suggests the need for targeted interventions to mitigate its effects. For instance, understanding, support and active listening—key findings in this metasynthesis—are necessary to build trust with patients and reduce the stigma associated with their condition.

Nurses should focus on educating patients and their caregivers about fibromyalgia and its impact, thereby promoting acknowledgment of the legitimacy of fibromyalgia symptoms. This approach aligns with findings indicating that legitimization of pain and illness experience can significantly reduce stigma. A future area of research is to examine caregiver behaviour toward chronic pain conditions and determine which behaviours are perceived as stigmatising. Further studies could explore how reducing stigmatising behaviours might impact the quality of care, improving patient outcomes and overall well‐being.

## 7 Limitations

This metasynthesis has several limitations. The topics were heterogeneous. Only two studies analysed and described stigma, while others investigated stigma indirectly. Another limitation is that although most of the studies met the methodological criteria according to the Joanna Briggs Institute checklist, the cultural/philosophical and theoretical position of the researcher and its influence on the research is rarely stated. Finally, querying only biomedical databases may have limited study of the phenomenon, especially from a psycho‐social perspective.

## 8 Conclusion

This metasynthesis highlights that stigma is a pervasive and multifaceted issue for individuals with fibromyalgia, deeply influencing their daily lives, social interactions, self‐esteem, and mental health. The findings underscore that stigma manifests in various forms, including workplace discrimination, challenges in accessing medical care and social judgement that undermines the legitimacy of patients' experiences. These insights emphasise the urgency of addressing fibromyalgia‐related stigma at multiple levels. Raising awareness and promoting education about fibromyalgia among health care professionals, caregivers and society at large are crucial steps toward reducing stigma and fostering a more supportive environment for patients. Awareness campaigns and educational programs can help dispel misconceptions, validate patients' experiences and encourage empathy and understanding.

Moreover, the results of this study underscore the need for health care professionals to adopt a holistic approach to fibromyalgia care. Beyond treating physical symptoms, professionals must recognise and address the psychosocial challenges patients face. This dual focus on physical and psychological well‐being could improve the quality of care, build trust between patients and providers and ultimately enhance patients' quality of life.

In conclusion, this study provides a foundation for future initiatives aimed at reducing stigma and improving the lives of individuals with fibromyalgia. Through collective efforts to increase awareness, combat discrimination, and promote inclusive care, it is possible to challenge societal biases and foster a culture of understanding and support for those living with this chronic condition.

## Author Contributions

**Benedetta Colombo:** conceptualization; data curation; formal analysis; investigation; writing – original draft preparation. **Eleonora Zanella:** conceptualization; data curation; formal analysis; investigation; writing – original draft preparation. **Alessandro Galazzi:** conceptualization; methodology; visualisation; writing – review and editing. **Paola Arcadi:** conceptualization; data curation; formal analysis; investigation; methodology; project administration; supervision; writing – review and editing.

## Conflicts of Interest

The authors declare no conflicts of interest.

## Supporting information


Data S1:

Data S2:

## Data Availability

Data derived from public domain resources.
