# Supplementary material for: The Experience of Stigma in People Affected by Fibromyalgia: A Metasynthesis
Source: J Adv Nurs. 2025 Jan 21;81(10):6317–32. doi: 10.1111/jan.16773 (PMC12460976; doi:10.1111/jan.16773)
Supplement: Supplementary file 1 — Data S1: [file JAN-81-6317-s001.docx]

Supplementary Table 1. Search strings

| **Database** | **Data** | **Query** |
| --- | --- | --- |
| PubMed | 25/06/23 | ((fibromyalgi*[Title/Abstract]) AND (stigma*[Title/Abstract] OR prejudice*[Title/Abstract] OR attitude*[Title/Abstract] OR discrimination*[Title/Abstract] OR stereotype*[Title/Abstract])) |
| CINAHL | 25/06/23 | (fibromyalgia or fibromyalgi*) AND (stigma* or prejudice or attitude* or discrimination or stereotype*) |
| Embase | 26/06/23 | fibromyalgia:ti,ab,kw AND stigma*:ti,ab,kw OR prejudice*:ti,ab,kw OR attitude*:ti,ab,kw OR discrimination:ti,ab,kw OR stereotype*:ti,ab,kw |
| Scopus | 26/06/23 | (TITLE-ABS-KEY (fibromyalgi*)) AND (TITLE-ABS-KEY (stigma* OR prejudice OR attitude* OR discrimination OR stereotype* ) |
| PsycInfo | 26/06/23 | (exp Fibromyalgia/ or fibromyalgia*.mp.) and (stigma* or prejudice* or attitude* or discrimination or stereotype*).mp. [mp=title, abstract, heading word, table of contents, key concepts, original title, tests & measures, mesh word] |

Legend: CINAHL, Cumulative Index to Nursing and Allied Health Literature.

Supplementary Table 2. Qualitative appraisal of the included studies

|  | **Item 1.** Is there congruity between the stated philosophical perspective and the research methodology? | **Item 2**. Is there congruity between the research methodology and the research question or objectives? | **Item 3**. Is there congruity between the research methodology and the methods used to collect data? | **Item 4**. Is the congruity between the research methodology and the representation and analysis of data? | **Item 5**. Is there congruity between the research methodology and the interpretation of results? | **Item 6.** Is there a statement locating the researcher culturally or theoretically? | **Item 7.** Is the influence of the researcher on the research, and vice- versa, addressed? | **Item 8**. Are participants, and their voices, adequately represented? | **Item 9**. Is the research ethical according to current criteria or, for recent studies, and is there evidence of ethical approval by an appropriate body | **Item 10.** Do the conclusions drawn in the research report flow from the analysis, ore interpretation, of the data? |
| --- | --- | --- | --- | --- | --- | --- | --- | --- | --- | --- |
| **Armentor, 2017** | Y | Y | Y | Y | Y | N | Y | Y | Y | Y |
| **Asbring & Närvänen, 2002** | Y | Y | Y | Y | Y | N | Y | Y | Y | Y |
| **Cunningham & Jillings, 2006** | Y | Y | Y | Y | Y | N | N | Y | Y | Y |
| **Escudero-Carretero et al., 2010** | Y | Y | Y | Y | Y | N | N | Y | Y | Y |
| **Henriksson, 1995** | Y | Y | Y | Y | Y | N | N | Y | Y | Y |
| **Juuso et al., 2011** | U | Y | Y | Y | N | Y | Y | Y | Y | Y |
| **Paulson et al., 2002** | Y | Y | Y | Y | Y | N | N | Y | Y | Y |
| **Peres, 2021** | Y | Y | Y | Y | Y | Y | Y | Y | Y | Y |
| **Rodham et al., 2010** | Y | Y | Y | Y | Y | N | N | Y | Y | Y |
| **Russell et al., 2018** | Y | Y | Y | Y | Y | Y | Y | Y | Y | Y |
| **Söderberg et al., 1999** | Y | Y | Y | Y | Y | Y | Y | Y | Y | Y |
| **Taylor et al., 2016** | Y | Y | Y | Y | Y | Y | N | Y | Y | Y |

Legend: Y= Yes, the article answered the qualitative evaluation question; N= No, the article did not answer the qualitative evaluation question; U= Unclear, the answer to the qualitative evaluation question is doubtful.
